# Short Communication: Conflicting Conclusions in PGT-A: Live Birth Rates per Initiated Cycle or Oocyte Retrieval Show No Benefit, but Significant Improvement per Embryo Transfer

**DOI:** 10.5935/1518-0557.20260055

**Published:** 2026

**Authors:** Isabela M. Pasotti, Claudia Petersen, Laura D. Vagnini, Fabiana C. Massaro, Bruna Petersen, Andreia Nicoletti, Juliana Ricci, Camila Zamara, Renata A. Pouza, Bianca C. Matuella, Elisangela V. Espirito-Santo, Joao B. Meziara, Antonio H Oliani, Joao Batista A. Oliveira, Jose G. Franco Jr

**Affiliations:** 1 Center for Human Reproduction Prof. Franco Jr, Ribeirão Preto, Brazil; 2 Paulista Center for Diagnosis - Research and Training, Ribeirão Preto, Brazil; 3 Department of Gynecology and Obstetrics, São José do Rio Preto School of Medicine (FAMERP), São José do Rio Preto, Brazil; 4 University of Beira Interior - Faculty of Health Sciences, Covilhã, Portugal; 5 Reproductive Medicine Unit - University Hospital Centre Cova da Beira, Covilhã, Portugal

**Keywords:** PGT-A, live birth rate, IVF, embryo transfer, initiated cycle

## Abstract

**Objective:**

To determine whether studies evaluating preimplantation genetic testing for aneuploidy (PGT-A) reach different conclusions depending on the denominator used to calculate live birth rates.

**Methods:**

A systematic review of studies published be-tween 2017 and 2025 was conducted using PubMed, Sco-pus, and Google Scholar. Randomized and non-randomized studies comparing IVF/ICSI cycles with and without PGT-A were included. Outcomes were analyzed per initiated cy-cle, oocyte retrieval, or embryo transfer. Relative risks were calculated using a random-effects model.

**Results:**

Eleven studies were included. No significant difference was observed when live birth rates were ana-lyzed per initiated cycle or oocyte retrieval (RR 1.04, 95% CI 0.91-1.18). However, PGT-A significantly improved live birth rates when analyzed per embryo transfer (RR 1.21, 95% CI 1.05-1.40).

**Conclusion:**

The apparent benefit of PGT-A depends on the denominator used. Reporting outcomes per embryo transfer introduces selection bias and overestimates treat-ment effectiveness. Clinically meaningful outcomes should be reported per initiated cycle or oocyte retrieval.

## INTRODUCTION

There are several ways to define success in assisted reproductive technologies (ART). Traditionally, researchers place the outcome in the numerator, such as ongoing pregnancy or live birth, and use as the denominator the number of oocyte retrievals or embryo transfers, but rarely the number of initiated cycles.

In some studies, the numerator refers simply to the presence or absence of a live-born infant, without a standardized definition regarding gestational age, derived from a single oocyte retrieval that may include outcomes from both fresh and frozen embryo transfers. In fact, there is no universal consensus regarding the optimal methodology to define success in ART. Even among major ART registries, such as ESHRE, SART, REDLARA, and ICMART, outcome metrics differ substantially (Fauser & Franx, 2026). These discrepancies may significantly influence the final medical interpretation of the effectiveness of any ART intervention. Preimplantation genetic testing for aneuploidy (PGT-A) has been proposed as a strategy to improve IVF/ICSI outcomes by selecting euploid embryos for transfer. Although several studies have suggested improved implantation and live birth rates with PGT-A, its universal clinical benefit remains controversial. One of the main methodological factors contributing to conflicting conclusions is the choice of denominator used to calculate live birth rates. Outcomes may be reported per initiated cycle, per oocyte retrieval, or per embryo transfer. Reporting outcomes per embryo transfer excludes cycles in which no embryos reach the transfer stage, thereby introducing selection bias and potentially overestimating treatment effectiveness.

Therefore, the objective of the present study was to evaluate whether comparative studies of IVF/ICSI cycles with or without PGT-A reach different conclusions depending on the denominator used to calculate live birth rates.

## MATERIALS AND METHODS

A comprehensive literature search was conducted in PubMed, Scopus, and Google Scholar covering publications from 2017 to 2025. The search included the keywords “PGT-A”, “morphological embryo selection”, and “live birth rate”. Randomized and non-randomized studies comparing IVF/ICSI cycles using PGT-A *versus* conventional morphological embryo selection (MES) were eligible. Studies were categorized into two groups according to the reported denominator: Group I included studies reporting live birth rates per initiated cycle or per oocyte retrieval, and Group II included studies reporting live birth rates per embryo transfer only.

### Statistical Methods

Data were extracted independently and analyzed using StatsDirect statistical software. Dichotomous outcomes were expressed as relative risk (RR) with 95% confidence intervals (CI). A random-effects model was applied to account for between-study variability. Statistical heterogeneity was assessed using Cochran’s Q test and the *I*^2^ statistic. Statistical significance was defined as *p*<0.05.

## RESULTS

A total of eleven studies met the inclusion criteria and were included in the quantitative analysis. In Group I, which included studies reporting live birth rates per initiated cycle or oocyte retrieval, no statistically significant difference was observed between PGT-A and conventional morphological embryo selection. The overall live birth rate was 54.7% (696/1273) in the PGT-A group and 54.0% (567/1050) in the control group (RR 1.04, 95% CI 0.91-1.18; *I*^2^=44.5%). See Table 1A.

**Table 1:** PGT-A vs Morphological Embryo Selection (MES): Live birth rates (LBR). 1A-Group I: LBR per initiated cycle or oocyte retrieval (CYI/OR). 1B-Group II: LBR per Embryo transfer (ET) only

1A-Group I: LBR per (CYI/OR)			
**Study**	**MES% (n/N)**	**PGT-A% (n/N)**	**RR (95% CI)**
Munné et al., 2019	50.3% (89/177)	41.9% (75/179)	0.83 (0.66-1.04)
Sato et al., 2019	26.0% (13/50)	35.7% (15/42)	1.37 (0.74-2.54)
Yan et al., 2021	62.1% (369/594)	66.3% (382/576)	1.07 (0.98-1.16)
Liu et al., 2024	41.9% (96/229)	47.1% (224/476)	1.12 (0.94-1.35)
**Total**	**54.0% (567/1050)**	**54.7% (696/1273)**	**1.04 (0.91-1.18)**
*χ^2^=0.31 (P=0.57); Q=5.12 (p=0.14); I^2^=44.5%*			
**1B**-Group II: LBR per ET only			
**Study**	**MES% (n/N)**	**PGT-A% (n/N)**	**RR (95% CI)**
Coates et al., 2017	63.1% (65/103)	78.2% (230/294)	1.24 (1.07-1.48)
Liss et al., 2018	23.5% (20/85)	49.5% (47/95)	2.10 (1.38-3.27)
Martello et al., 2021	45.5% (10/22)	59.1% (13/22)	1.30 (0.74-2.37)
Namath et al., 2021	40.4% (157/389)	41.2% (80/194)	1.02 (0.83-1.25)
Zhou et al. 2021	40.7% (66/162)	45.4% (54/119)	1.11 (0.85-1.46)
Awadalla et al., 2022	52.5% (53/101)	70.0% (49/70)	1.33 (1.04-1.70)
Pantou et al., 2022	21.9% (61/279)	21.6% (38/176)	0.99 (0.69-1.41)
**Total**	**37.9% (432/1141)**	**52.7% (511/970)**	**1.21 (1.05-1.40)**

*χ^2^=7.11 (P=0.008); Q=11 (p=0.083); I^2^=46.3%*

In contrast, Group II studies reporting outcomes per embryo transfer demonstrated a statistically significant improvement in live birth rates with PGT-A. The overall live birth rate was 52.7% (511/970) in the PGT-A group compared with 37.9% (432/1141) in the control group (RR 1.21, 95% CI 1.05-1.40; ***I***^2^=46.3%). See Table 1B.

## DISCUSSION

Recent position statements from major reproductive medicine societies have raised important concerns regarding the routine use of PGT-A. The Practice Committees of the American Society for Reproductive Medicine and the Society for Assisted Reproductive Technology (2024). noted that although the use of PGT-A has increased and the underlying technologies continue to evolve, its value as a routine screening test for all IVF patients has not been demonstrated. While earlier single-center studies suggested higher live birth rates among patients with a favorable prognosis, more recent multicenter randomized controlled trials have reported similar outcomes between PGT-A and conventional IVF when frozen embryo transfer is performed. The value of PGT-A in reducing clinical miscarriage also remains uncertain. Similarly, the European Society of Human Reproduction and Embryology concluded that currently available data show only limited improvement in live birth rates with PGT-A, and that claims of reduced miscarriage rates or shorter time to pregnancy in specific subgroups require further validation (ESHRE Add-ons working group, 2023).

Despite these cautious recommendations, the use of PGT-A continues to increase worldwide. The findings of the present study may help explain why conclusions in the literature appear contradictory. The perceived effectiveness of PGT-A depends heavily on the denominator used to calculate live birth rates. When outcomes are reported per initiated cycle or per oocyte retrieval-metrics that more accurately reflect the patient experience and treatment burden-PGT-A does not improve live birth rates compared with conventional embryo selection (Munné *et al*., 2019; Sato *et al*., 2019; Yan *et al*., 2021; Liu *et al*., 2024). However, when outcomes are reported per embryo transfer only, PGT-A appears to confer a significant benefit (Coates *et al*., 2017; Liss *et al*., 2018; Martello *et al*., 2021; Namath *et al*., 2021; Zhou *et al*., 2021; Awadalla *et al*., 2022; Pantou *et al*., 2022).

This apparent benefit is largely attributable to selection bias, as cycles without transferable embryos-either because no embryos reach the blastocyst stage or because all embryos are classified as aneuploid-are excluded from the analysis. Consequently, reporting outcomes per embryo transfer artificially enriches the analyzed population with patients of more favorable prognosis. Clinically meaningful outcome measures should reflect the probability of live birth per initiated treatment, as this represents the true effectiveness of IVF from the patient’s perspective.

The lack of standardized reporting contributes substantially to the ongoing controversy regarding PGT-A. Until consistent and unbiased outcome definitions are adopted, conflicting conclusions regarding its clinical utility will persist. Future studies should prioritize reporting live birth rates per initiated cycle and per oocyte retrieval. From both a mathematical and clinical standpoint, current evidence does not support a universal benefit of PGT-A when evaluated using unbiased denominators. Reproductive medicine societies should define, with greater precision, which metrics best represent success in ART.

## CONCLUSION

The apparent benefit of PGT-A depends on the denominator used. Reporting outcomes per embryo transfer introduces selection bias and overestimates treatment effectiveness. Clinically meaningful outcomes should be reported per initiated cycle or oocyte retrieval.
